# Supplementary material for: Low-volume enrichment method supports high throughput bacteriophage screening and isolation from wastewater
Source: PLoS One. 2024 Apr 16;19(4):e0298833. doi: 10.1371/journal.pone.0298833 (PMC11020952; doi:10.1371/journal.pone.0298833)
Supplement: Attachment — Submitted filename: Response to Reviewers.docx [file pone.0298833.s004.docx]

**Response to Reviewers**

Journal requirements:

*1. When submitting your revision, we need you to address these additional requirements.*

*Please ensure that your manuscript meets PLOS ONE's style requirements, including those for file naming. The PLOS ONE style templates can be found at*

*https://journals.plos.org/plosone/s/file?id=wjVg/PLOSOne_formatting_sample_main_body.pdf and*

[*https://journals.plos.org/plosone/s/file?id=ba62/PLOSOne_formatting_sample_title_authors_affiliations.pdf*](https://journals.plos.org/plosone/s/file?id=ba62/PLOSOne_formatting_sample_title_authors_affiliations.pdf)*.*

**Response:** Concur with editor comment. We have read guidelines and made appropriate adjustments to the manuscript to adopt PLOSOne’ style requirements.

*2. We note that your Data Availability Statement is currently as follows: [All relevant data are within the manuscript and its Supporting Information files.]*

*Please confirm at this time whether or not your submission contains all raw data required to replicate the results of your study. Authors must share the “minimal data set” for their submission. PLOS defines the minimal data set to consist of the data required to replicate all study findings reported in the article, as well as related metadata and methods (https://journals.plos.org/plosone/s/data-availability#loc-minimal-data-set-definition).*

*For example, authors should submit the following data:*

*- The values behind the means, standard deviations and other measures reported;*

*- The values used to build graphs;*

*- The points extracted from images for analysis.*

*Authors do not need to submit their entire data set if only a portion of the data was used in the reported study.*

*If your submission does not contain these data, please either upload them as Supporting Information files or deposit them to a stable, public repository and provide us with the relevant URLs, DOIs, or accession numbers. For a list of recommended repositories, please see https://journals.plos.org/plosone/s/recommended-repositories.*

**Response:** Concur with editors. We have created a data set that includes information used to create figures and tables. This dataset has been uploaded at DOI 10.17605/OSF.IO/AHGJF for open access. This documentation is in addition to information already available in the manuscript, tables and figures.

*3. We note that Figure 1 and 2 in your submission contain copyrighted images. All PLOS content is published under the Creative Commons Attribution License (CC BY 4.0), which means that the manuscript, images, and Supporting Information files will be freely available online, and any third party is permitted to access, download, copy, distribute, and use these materials in any way, even commercially, with proper attribution. For more information, see our copyright guidelines: http://journals.plos.org/plosone/s/licenses-and-copyright.*

*We require you to either (1) present written permission from the copyright holder to publish these figures specifically under the CC BY 4.0 license, or (2) remove the figures from your submission:*

*a. You may seek permission from the original copyright holder of Figure 1 and 2 to publish the content specifically under the CC BY 4.0 license.*

*We recommend that you contact the original copyright holder with the Content Permission Form (http://journals.plos.org/plosone/s/file?id=7c09/content-permission-form.pdf) and the following text:*

*“I request permission for the open-access journal PLOS ONE to publish XXX under the Creative Commons Attribution License (CCAL) CC BY 4.0 (http://creativecommons.org/licenses/by/4.0/). Please be aware that this license allows unrestricted use and distribution, even commercially, by third parties. Please reply and provide explicit written permission to publish XXX under a CC BY license and complete the attached form.”*

*Please upload the completed Content Permission Form or other proof of granted permissions as an ""Other"" file with your submission.*

*In the figure caption of the copyrighted figure, please include the following text: “Reprinted from [ref] under a CC BY license, with permission from [name of publisher], original copyright [original copyright year].”*

**Response:** We concur with editors. Figure 1 and Figure 2 were designed and built using an application designated BioRender. Patrick Kenney, co-author of this manuscript, has been granted a license to use the BioRender application, including its content, icons, templates and other original artwork. Accordingly, Figures 1 and 2 now have appropriate release documentation for publication that is added to the resubmission. According to BioRender, this license permits BioRender content to be sublicensed for use in journal publications. All rights and ownership of BioRender content are reserved by BioRender. All completed graphics are accompanied by this citation: “Created with BioRender.com”. BioRender content included in the completed graphic is not licensed for any commercial uses beyond publication in a journal.

The figure legends in the initial submission were removed from all of the *.tiff files to ensure there was only one copy of the legend and avoid that way accidental inconsistency. Figure legends are included at the end of the main manuscript.

*4. Please review your reference list to ensure that it is complete and correct. If you have cited papers that have been retracted, please include the rationale for doing so in the manuscript text, or remove these references and replace them with relevant current references. Any changes to the reference list should be mentioned in the rebuttal letter that accompanies your revised manuscript. If you need to cite a retracted article, indicate the article’s retracted status in the References list and also include a citation and full reference for the retraction notice.*

**Response:** We have reviewed the references list and we confirm that all information is accurate.

Reviewers' comments:

***Reviewer #1****: The authors describe a method for rapid phage isolation with fewer resource requirements relative to traditional methods. Though less sensitive, the method could be helpful in several circumstances, particularly when time is a concern. In general, the paper is well-written and the methodology used is sound. Though isolating phages in such a manner has been previously reported (using 96-well plates and observing growth curves for inhibition), the authors study adds value by evaluating a wide range of species and assessing several different phage sources. Additionally, they provide a time and cost analysis, which is helpful. I would also note that their results are consistent with many of our own findings in regard to the isolation of phages from wastewater, so I would imagine reproducibility would be high. Overall, the reported method is likely to be frequently adopted by other research groups.*

**Response:** The authors thank this reviewer for the thorough and thoughtful comments – they are much appreciated!

***L46****: Many would argue that this was not the first clinical use of personalized phage therapy, though it is certainly one of the more high-profile cases in the US.*

**Response:** Concur with reviewer comment. Accordingly, the sentence in question: “The first clinical use of personalized phage therapy was in the treatment of a multidrug-resistant Acinetobacter baumannii infection in 2016” was changed to read: “A highly popularized clinical use of personalized phage therapy was in the treatment of a multidrug-resistant Acinetobacter baumannii infection in 2016”

***L77****: Should report time of year when wastewater was collected. We've found it can have significant impact of the success of phage isolation.*

**Response:** Appreciate reviewer comment. Accordingly, Line 77 was adjusted to read, “In April 2023, a local wastewater treatment facility…”

***L99****: Though it is reported a few paragraphs before, I would suggest saying "filtered wastewater" to make it more obvious to the reader.*

**Response.** Agree with reviewer. Accordingly, we have edited the phrase to read: “filtered wastewater”

***L106****: Do you mean 0.7% agar?*

**Response:** Appreciate reviewer recognition of typo. The answer is Yes. The phrase was edited and it now reads: “0.7% agar”, not 70%.

***L134****: Sentence is a bit confusing. Meaning assessing the negative samples via conventional plaque assay?*

**Response:** Appreciate reviewer comment. Accordingly, we edited the sentence. The sentence in question: “At a species level, only coagulase-negative *Staphylococcus*, *Vibrio cholerae*, and *Yersinia enterocolitica* did not have lytic phage detected during testing,” was change to: “Coagulase-negative *Staphylococcus*, *Vibrio cholerae*, and *Yersinia enterocolitica* were the only species without plaques found in conventional plaque assays.”

***L143****: aureus?*

**Response:** This isolate was a non-*Staphylococcus aureus*. Conventional microbiologic testing did not differentiate the species further, so it is in our biobank simply as “Coagulase-negative *Staphylococcus*”

***L151****: The traditional and low-volume growth conditions seem to have only significantly differed by volume (6 mL vs 200 ul enrichments). Any hypotheses as to why this makes a difference for isolate growth? Perhaps gas exchange?*

**Response:** Appreciate reviewer question. Although, we have no experimental evidence regarding oxygen levels of culture environments with the traditional vs low-volume methods, we agree with reviewer that differential gas-exchange may have affected bacterial growth. In fact, the isolates that had poor growth were more anaerobic in nature. Therefore, the impairment of gas exchange at the lower parts of the test tubes, compared to a shallow wells of 96-well plates, likely promoted growth of those species. The differences in the low volume samples between DI and RAS are less clear and likely related to some undefined inhibitory components in the RAS. A note to this regard was placed in this section of the text.

***L168:*** *We generally sample the AS directly, and not the RAS. Some additional details of what this type of sample is like may be helpful. Also, were any steps taken to elute phages from the solids? I would expect fairly different microbial (and therefore phage) compositions between DI and AS.*

**Response:** Appreciate reviewer comment. Obtaining RAS vs AS was facilitated by the water plant facility and the processing of both samples was out of interest scientific curiosity. We did intentionally obtain both RAS and DI to allow for greater microbial diversity. In particular, aerobes are only rarely isolated in RAS compared to DI. We did not use elution steps, but we did ensure that solids were present in solution right up until the point of centrifugation/filtration for a day’s experiments to hopefully allow for better capture. A sentence regarding differences between RAS and DI was added to the text.

***L201****: I'm glad the authors mention the use of concentration methods to improve yield, but evaluating this would have been easy and added much value to the study.*

**Response:** We thank reviewer for the comment. Concentration methods would have been ideal, and it was in fact attempted. Unfortunately, time would not allow for concentration steps. We did not have an equipment setup for TFF or ultracentrifugation, and our suppliers were quoting multi-month delays for ultrafiltration tubes in the post-COVID supplies crunch.

***Table 2:*** *The number of samples is somewhat confusing between the low-volume and traditional methods. For traditional, 66 isolates x 4 phage sources = 264 combinations. For the low-volume, 8 plates x 96 wells = 768 wells. Were triplicate wells used? If so, 768/3 = 256 samples.*

**Response:** 8 plates is an artifact of the amount of effort involved in simultaneous setup; I found that trying to manage more than 16 bacterial isolates in the traditional method was too labor intensive, so I limited each plate to 16 bacterial isolates and 2 waste conditions.

***Table 4:*** *For some samples (e.g., Bordetella), phages were found for the low-volume but not traditional methods. Why might that be since the traditional is typically more sensitive (as the authors report)?*

**Response:** Appreciate reviewer question. We do not have a good explanation for this finding. One possible explanation with no experimental evidence is that low-volume conditions increase environmental stress of *Bartonella* which may result in upregulation of genes mediating lytic cycle of Bordetella-associated phages. Traditional method, using larger liquid media volume and less environmental stress, may downregulate genes mediating lytic cycle of Bordetella-associated phages rendering no free phages to be detected in media.
